# An Atmospheric Chemistry Perspective on Airborne Micro- and Nanoplastic Particles

**DOI:** 10.1021/acs.est.5c03264

**Published:** 2025-04-14

**Authors:** Yue Zhang, Jonathan H. Slade, Andrew P. Ault, Arthur W. H. Chan

**Affiliations:** † Department of Atmospheric Sciences, Texas A&M University, College Station, Texas 77843, United States; ‡ Department of Chemistry and Biochemistry, University of California, San Diego, La Jolla, California 92093, United States; § Department of Chemistry, University of Michigan, Ann Arbor, Michigan 48109, United States; ∥ Department of Chemical Engineering & Applied Chemistry, University of Toronto, Toronto, Ontario M5S 3E5, Canada

**Keywords:** microplastics, nanoplastics, atmosphere, aerosols, transformation

## Abstract

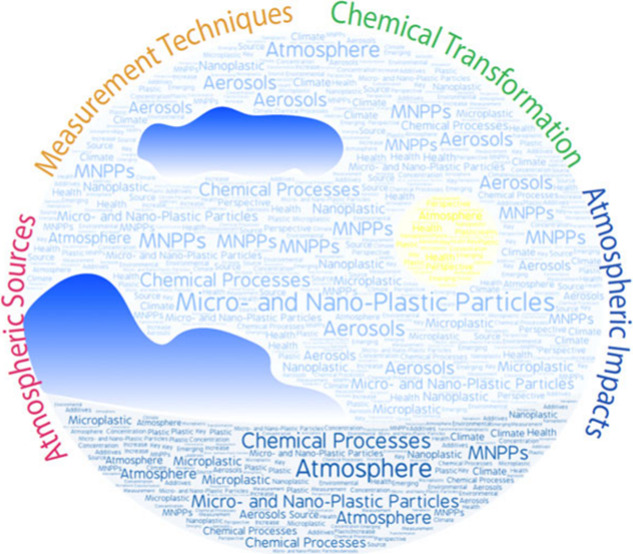

Micro- and nanoplastic particles (MNPPs) are emerging pollutants with significant environmental impacts due to their persistence, increasing concentrations, and potential health risks. Most MNPP studies have focused on identifying, quantifying, and assessing their ecotoxicological impacts in water or soil. However, the atmosphere is crucial in transporting and chemically transforming MNPPs. Further, well-established aerosol particle characterization techniques are underutilized and inconsistently applied in existing atmospheric MNPP studies. This perspective synthesizes the existing literature and addresses future research needs unique to atmospheric MNPPs, highlighting the need to bridge the microplastics and atmospheric aerosol communities to better understand their sources, chemical transformations, transport mechanisms, as well as their health effects and ecological impacts, which differ from those in soil and water. Advancing research in these areas requires standardized methods and a multidisciplinary approach to comprehensively assess MNPP interactions across environmental compartments, providing essential insights into their environmental fate and risks.

## Abstract 2

This perspective outlines the current research and provides recommendations for future research on emerging atmospheric MNPPs, bridging a key gap in understanding the processes and impacts of MNPPs in environmental systems.

## 1 Introduction

Plastics, operationally defined as manufactured materials made of repeating polymer units, are widely used for their versatility, flexibility, and low cost.^1−4^ Global plastic production is exponentially increasing, reaching nearly 460 million metric tons (Mt) in the year 2019 alone.^5^ This represents a reservoir of materials in the environment that is seven times the annual global production rate of secondary organic aerosol (SOA).^6^ Cumulatively, 9200 Mt of plastics have been produced globally as of 2019.^5^ About 700 Mt or 7.6% of all plastics ever made has been recycled, while 57.6% has been discarded.^4,7^ The remaining plastics are either in use (23.9%) or incinerated (10.9%).^4,5^ Plastics degrade over time through mechanical, thermal, photochemical, and biochemical mechanisms.^8^ Some break down into microplastic particles (MPPs) and nanoplastic particles (NPPs),^9,10^ with a size of 1 μm to 5 mm and less than 1000 nm, respectively.^11,12^ Micro- and nanoplastic particles (MNPPs) are also manufactured (e.g., as micro- or nanobeads, pellets, and nanotubes) and directly enter the environment.^13^

**Figure 1:**
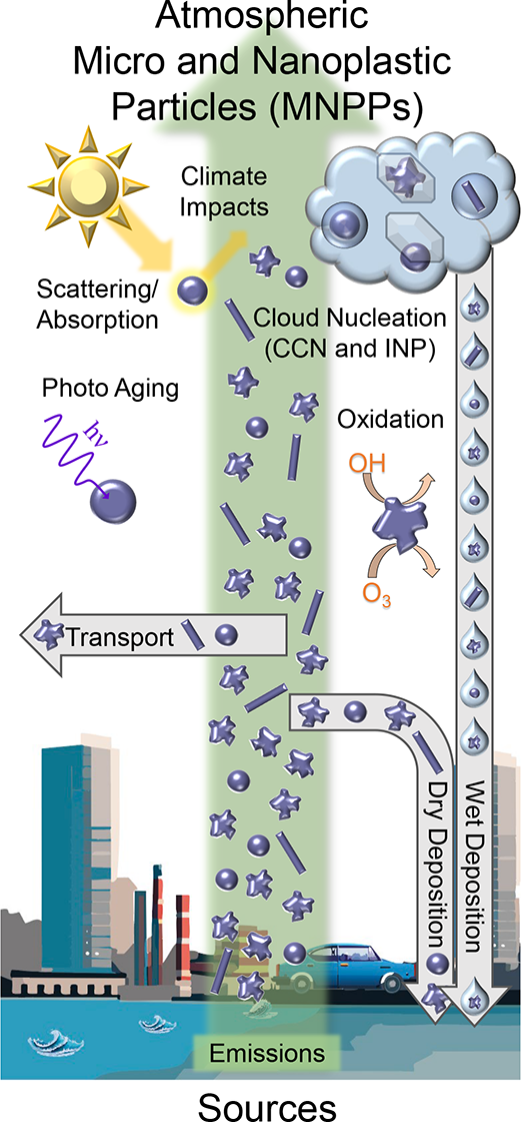
Overview of the physical and chemical processes related to atmospheric MNPPs.

MNPPs have now been found in all environmental compartments: the marine environment,^14−16^ freshwater,^17^ land and soil,^18^ as well as the atmosphere, as shown in Figure 1.^19^ Among these, the atmosphere plays a crucial role in the transport and fate of MNPPs. Studies have collected MNPPs from the atmosphere in remote areas,^20,21^ highlighting the atmosphere’s key role in transporting plastic particles globally.^22^ Plastic particles suspended in air are more mobile than in other environmental systems, making atmospheric transport a major pathway for the global movement of plastic materials from sources to distant areas.^23−27^ Once airborne, MNPPs undergo physical and chemical aging processes, which can impact health and climate differently than in other environments,^28,29^ underscoring the importance of examining MNPPs from an atmospheric perspective.

This perspective addresses the sources, concentrations, and chemical processing of atmospheric MNPPs, as well as their broader implications for health and climate. Unlike existing reviews that predominately focus on the toxicology of MNPPs or their identification in water and soil, this article emphasizes the distinct challenges and opportunities in studying MNPPs in the atmosphere. We highlight the differences between MNPPs in air versus water, particularly the significance of size on atmospheric lifetime and transformations, and address key terminology distinctions such as primary versus secondary particles. This perspective also explores the unique atmospheric modifications and interfacial chemistry of MNPPs, including oxidation processes, interactions with sunlight, and their potential roles as cloud condensation nuclei (CCN) and ice nucleating particles. In addition, differences between prior work on MPPs and NPPs and the needs of atmospheric studies are discussed due to the importance of understanding the role of size in atmospheric aerosols. By leveraging well-established atmospheric sampling and analytical techniques that have been underutilized in MNPP research, this article aims to bridge the microplastics and atmospheric aerosol communities to address future research needs on atmospheric MNPPs, offering a comprehensive viewpoint that has not been elaborated upon in prior studies.

## 2 MNPP Sources and Concentrations

### 2.1 Potential Atmospheric Sources

Polymer degradation is a major source of MNPPs.^30^ and their environmental lifetimes depend critically on their chemical, mechanical, and physical properties.^1−6,31,32^ Plastics often contain chemical additives like hardeners, ultraviolet filters, antimicrobials, and flame retardants to extend their lifetimes. Consequently, plastics are often stable in the environment for an extended time, accumulating in environmental systems for decades to possibly millennia time scales.^31,33^ Degradation of large plastic debris, transport from the terrestrial and aquatic environments, and direct emissions from industrial activities are major sources of atmospheric MPPs,^34^ though the contribution from each source remains unclear.^35^ There have been some studies showing that heating plastic material during pipe curing,^36^ burn pit smoke and facemasks,^37,38^ trash incineration,^39,40^ and three-dimensional (3D) printing^41^ can lead to the production and emissions of NPPs in air, but their atmospheric burdens are poorly understood.

A potentially significant burden of atmospheric MNPPs comes from air-sea exchange.^42,43^ Recent modeling estimates an astounding 3200 kilotons of ocean plastic pollution, encompassing plastics on the surface, in the deep sea, and along beaches,^44^ and sets an upper limit for the potential emission of MNPPs in the marine atmospheric environment as these plastics break down. Gigault et al. suggested that the breakdown of ocean microplastics would result in 5 quadrillion (10^15^) NPPs.^33,45^ The smaller size of NPPs increases their propensity to enter the atmosphere,^13,46^ such as through wave-breaking and bubble-bursting processes that eject nanosized and micrometer-sized particles accumulated at the ocean surface.^47^ Bottom-up estimates of this atmospheric flux are based on laboratory aerosolization of seawater, but the efficiency of aerosolizing MNPPs depends on plastic-type,^48^ the extent of ultraviolet (UV) aging, and the presence of surfactants.^49^ The range of bottom-up estimates is much lower than top-down estimates,^48,50−52^ suggesting an incomplete understanding of the emission mechanisms or insufficient observations to accurately back-calculate emission rates.^8,11−13,33,45,46^

### 2.2 Implications of MNPP Size on Transport and Impacts

**Figure 2:**
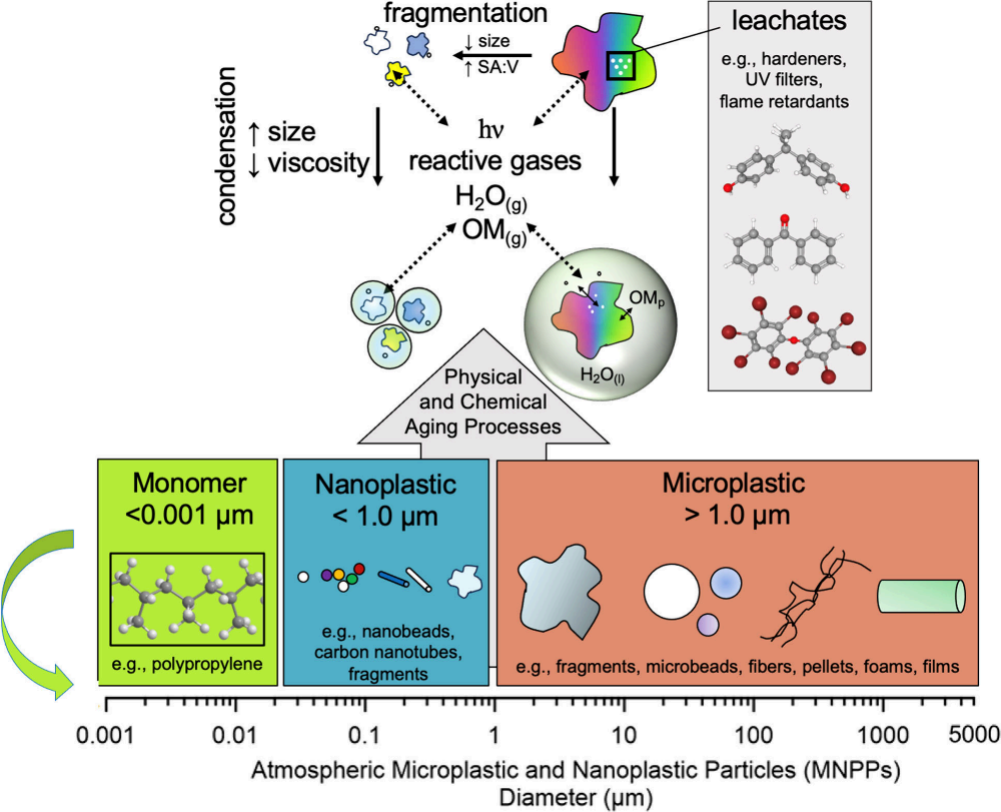
Schematics of atmospheric aging processes of MNPPs with different size ranges.

Previous studies have outlined the critical differences between MPPs and NPPs, including composition, mass transfer rates of additives, surface interactions, and potential health impacts.^8,11−13^ In the atmospheric context, there are additional considerations regarding the physical and chemical behaviors of super- and sub-micrometer particles, as indicated in Figure 2. Aerosols larger than 2.5 μm but smaller than 10 μm are often referred to as coarse particles. Since mass scales proportionally to particle diameter cubed (*m* ∝ *d*_p_^3^), MPPs in the coarse particle size range may contribute disproportionately to higher mass concentrations than fine particles (*d*_p_ < 2.5 μm), even when their number concentrations are lower. The larger diameters of coarse MPP lead to faster terminal settling velocities compared to fine particles (*v*_TS_ ∝ *d*_p_^3^), resulting in shorter atmospheric lifetimes.^53^

The smaller sizes of NPPs, especially those in the ultrafine particle (*d*_p_ < 0.1 μm) size range may exhibit faster Brownian diffusion and coagulation rates. The larger surface areas of NPPs also allow for greater gas-to-particle transfer of semivolatile organic and inorganic compounds. The smaller size of the NPPs lead them to deposit deeper into the respiratory tract than super-micrometer particles,^54^ as sub-micrometer particle can reach the alveoli of the lungs^55^ or cross the blood–brain barrier via the circulatory system.^56^ The above evidence suggests that NPPs in the air will likely have longer atmospheric lifetimes, age differently, and potentially pose greater health impacts than MPPs.

Atmospheric circulation is an important pathway for global MNPP transport.^57^ The inland transport of airborne plastics is significant as more than 1000 tons of plastic are deposited in protected lands in the western U.S. annually.^52^ In addition, approximately 1.2 tons of small microplastics are transported to the marine environment through the atmosphere in the western Pacific Ocean annually.^58^ Settling time scales of spherical particles greater than 1 μm are generally days or less, suggesting the importance of MPPs for local or regional deposition. However, if lofted, MPPs could be transported further like mineral dust, which has been shown to circumnavigate the globe.^59^ Meanwhile, NPPs are likely more relevant for atmospheric transport over longer ranges. For example, a significant fraction of brake wear plastic particles lie in the sub-micrometer size range and can deposit in remote regions.^57^ Water uptake by MNPPs is generally ineffective,^60^ implying slow removal by cloud nucleation and a longer atmospheric lifetime than other aerosols.^61^ However, plastics are viscous, and viscous particles are efficient at templating ice.^62−64^ A recent study suggests MPPs are a non-negligible source of ice nucleating atmospheric particles.^65^

## 3 Atmospheric Techniques for Quantification of MNPPs

Accurately measuring and reporting atmospheric MNPP concentrations is essential for understanding their emissions and atmospheric modifications. A wide range of sampling techniques have been adopted to collect atmospheric MNPPs, including deposition sampling, rain/snow collection, active sampling (filtration or impaction) using a pump, and direct introduction for analysis (such as an aerodynamic lens). Numerous chemical analysis techniques have been used to analyze atmospheric MNPPs offline, with the most commonly used including microscopy, spectroscopy, and mass spectrometry.^2^ However, a challenge posed by the relatively recent emergence of the MNPPs research area is the lack of standardized sampling and analysis methods to quantify airborne MNPPs,^10^ resulting in wide variations in their physical (size), chemical, and temporal resolution.^9,66^

Early analysis of atmospheric MPPs was based on optical microscopy, which probed particles for distinctive shape, morphology, and color to identify them as plastics.^17^ While essential in initially identifying atmospheric MPPs, these methods were often biased toward particles of larger sizes, which may be less important due to their short atmospheric lifetimes.^53^ For smaller NPPs, optical microscopy may face limitations in confidently distinguishing plastics from other materials. Scanning electron microscopy (SEM) and transmission electron microscopy (TEM) were also used, but they faced similar challenges in confidently identifying MPPs within complex atmospheric samples due to limited chemical information. Fluorescence microscopy, including staining samples with Nile Red dye, emerged to identify smaller MNPPs optically but struggled with false positives, lengthy workflows, and challenges in quantitatively determining atmospheric concentrations.^67^ To overcome these limitations, *in situ* fluorescence techniques have been recently developed to quantify various types of airborne MPP in real-time, achieving high detection efficiency for micrometer-sized plastic particles.^68,69^

A unique identifying feature of plastics during analysis is their polymeric composition, which must be differentiated from other substances in the atmosphere.^70,71^ The desire for chemical information, in addition to image analysis, has led to using many common spectroscopic techniques for polymer characterization, focusing on infrared (IR) and Raman spectroscopy. Raman microspectroscopy,^72,73^ can analyze far smaller particles (practically down to ∼700 nm) using visible light (often 532 or 785 nm). A limitation of Raman analysis is autofluorescence, which can be mitigated by photobleaching or using longer wavelengths (with worse spatial resolution).^74^ However, certain MNPPs in environmental and atmospheric samples still fluoresce.^73^ These vibrational techniques (IR and Raman) have extensive libraries used in the polymer industry, and the distinctive spectra of plastic polymers can be confidently identified even in complex mixtures. Since microscopy may not be able to differentiate atmospheric MNPPs from other particles purely based on shapes, leading to a focus on microscopy coupled with spectroscopy that includes information on chemical composition as well, namely microspectroscopy (microscopy + spectroscopy).^71^ However, microspectroscopy can have long analysis times (∼100 particles per hour) leading to lower throughput.

Multiple IR microscopy methods have been applied to the study of MPPs, such as Fourier transform infrared (FTIR) microscopy^75^ and more advanced methods using focal plane arrays (FPA)^76^ or quantum cascade lasers (QCLs).^77,78^ IR microscopy can identify specific plastics and not just MNPPs in general. However, the diffraction limit of IR radiation results in poor spatial resolution, which makes analyzing atmospheric particles <5–10 μm virtually impossible.

A pump–probe IR microspectroscopy method, optical photothermal infrared spectroscopy (O-PTIR), can overcome the spatial limitations of FTIR microspectroscopy and fluorescence issues of Raman microspectroscopy.^79^ O-PTIR uses the photothermal effect by scanning across the mid-infrared with a tunable QCL. When a particle has an IR absorption mode, it heats up and expands, along with experiencing a slight shift in the refractive index. These effects combine to increase the scattering of a co-located continuous-wave visible laser (532 nm).^80^ O-PTIR achieves the same spatial resolution as Raman microspectroscopy (<1 μm), while obtaining IR and Raman spectra simultaneously for further identification.^81^ O-PTIR+Raman has been increasingly used to study MPPs in recent years.^81,82^

An alternative to microspectroscopy for identifying MNPPs is the use of mass spectrometry. The benefits of mass spectrometry include the ability to conduct analysis for confident identification in complex mixtures rapidly and to analyze nanosized materials. Multiple mass spectrometry methods have been used to quantify airborne MPPs and have been extensively reviewed in other works.^83,84^ Though not an exhaustive list, these include offline analyses such as pyrolysis gas chromatography mass spectrometry (Py–GC–MS),^85,86^ and thermal desorption proton transfer reaction mass spectrometry (TD–PTR–MS),^87^ as well as online techniques such as high-resolution time-of-flight aerosol mass spectrometry (HR-ToF-AMS),^39,88^ which can be used for either MPPs or NPPs depending on the sampling collection method. The constituent polymers or polymer fragments are then ionized and detected based on their mass-to-charge ratio. These tools are highly sensitive and have detection limits of polymers in complex matrices of nanograms per cubic meter of air over relatively short time scales.^39,86,89,90^ One drawback is that these techniques are destructive and require sufficient sample mass followed by heating to temperatures high enough for thermal desorption or pyrolysis.^84^ Furthermore, plastic molecular fragments may not be specific to any one type of plastic and mass spectrometry quantifies based on mass, not number. Therefore, distinguishing airborne plastics based on a single tracer ion or determining how many MNPPs are present in a sample is still an ongoing effort. It is worth noting that quality control and quality assurance procedures are especially important for quantifying MNPPs, due to their low concentration in the air. Internal standards or cross comparisons with different techniques are desirable to ensure high quality data for modeling and policy purposes. In addition, improved metrics for positive identification of microplastics when comparing to standards and spectral libraries are needed and being developed.^120^

## 4 MNPP Chemical Processes

### 4.1 Atmospheric Aging and Potential Products

Physical and chemical aging are two major pathways for atmospheric processing of plastics.^91,92^Figure 2 shows how UV light and oxidants can age plastics through chemical reactions.^92^ During aging, both physical (e.g., size, shape and morphology, density, phase state, and refractive index) and chemical properties (e.g., composition, hygroscopicity, acidity, solubility, and oxidation state) change. This can lead to rougher surfaces, more oxygen-containing functional groups present, and a higher adsorption capacity.^93^

Due to the low hygroscopicity of MNPPs, many of the reactions involving the aging of these plastic particles are likely multiphase reactions, where gas or liquid phase oxidants react at the interface between the plastic and the medium. Multiphase chemical systems have been widely studied in atmospheric science to examine interfacial interactions. In addition, secondary MNPPs can be generated from these processes, from the break down of large plastic debris.^94,95^ Plastic chemical additives, including bisphenol A (hardener) and oxybenzone (UV filter),^96^ can also lead to different aging rates in different environmental matrices.^97^ While the atmospheric chemistry, kinetics, and products of these multiphase reactions with MNPPs caused by exposure to ultraviolet rays, heat, and biological processes are still unclear, their toxicity and lifetimes may be modified by atmospheric aging processes and differ depending on if they are suspended as an aerosol or in bulk water.^96^ These reaction processes also impact the number and concentration of plastic particles and their interactions with clouds and radiation.^58,98^

### 4.2 Surface Adsorption of MNPPs

MNPPs have been shown to adsorb organic pollutants due to their surface structure,^99^ including per- and polyfluorinated species (PFAS) and persistent organic pollutants (POPs).^100,101^ There are multiple factors affecting the adsorption process, such as MNPP characteristics (e.g., composition/type, structure, binding energy, and surface properties), release medium (e.g., pH, temperature, salinity, and ionic strength), and contamination factors (e.g., solubility, redox state, charges, and stability). In addition, the adsorption properties of MNPPs have been shown to increase with aging.^93^

## 5 Impacts of Atmospheric MNPPs

### 5.1 Impacts on Climate

The climate impacts of aerosols include both direct and indirect effects. Direct forcing involves aerosol scattering and absorbing radiation, while indirect forcing refers to the ability of aerosols to alter cloud formation and cloud properties, which can in turn alter solar radiation. MNPPs are estimated to have a net negative direct forcing of 0.044 mW m^–2^.^102^ However, this forcing may increase further if MNPP concentrations increase rapidly.^103^ MNPPs could play a more significant role in indirect forcing, as MNPPs are potential ice nucleating particles (INPs) both in the immersion freezing and deposition freezing regimes from recent studies.^65,104,105^ Currently, the number concentrations of MNPPs are still relatively low in the free troposphere,^102^ but are expected to increase substantially and potentially play a more important role in ice nucleation, especially in areas with higher MNPP concentrations.

### 5.2 Impacts on Human Health

It is estimated that humans inhale tens of thousands of MNPPs daily.^106^ The concentrations of MNPPs in indoor environments, where people spend most of their time, are about an order of magnitude greater than in outdoor air.^107^ Previous studies state that the most notable routes of MNPP exposure to humans are through inhalation, ingestion, and dermal sorption through the use of personal care products (PCPs) containing MNPPs,^108^ with other exposure pathways such as deposition and exposure through contact on skin and food surfaces.^101^ Once MNPPs enter the human body, they can potentially translocate from the exposed organ to other body compartments, leading to inflammation.^101^ In addition, as particle size decreases, the surface area to mass ratio increases, increasing the likelihood of subsequent interactions with biological barriers.^108,109^ In addition, MNPPs may also cause dyspnea, as well as airway and interstitial lung diseases in industries involving flock, synthetic textiles, and PVC,^101,110−113^ suggesting that susceptible individuals may be at risk of developing similar lesions even when environmental concentrations are low.^114^ The smaller size of NPPs also allow faster and more efficient transport of plastic particles to the brain, leading to potentially more significant adverse health effects.

### 5.3 Impacts on Ecology

MNPPs can negatively impact surface plants. The phyllosphere is the largest interface between the atmosphere and terrestrial ecosystems and is a major sink for MNPPs.^115^ Atmospheric MPPs can absorb onto leaves, with an abundance of 3.62 ± 1.29 items cm^–2^.^116^ These MNPPs were mainly below 80 μm, dominated by polyamide, polyethylene, and rubber,^117^ and likely impacted the structure and functions of the phyllosphere bacterial community (PBC).^116^ In addition, functions related to human diseases and cellular processes have been positively correlated with microplastic size distribution,^118^ highlighting the ecological risks of atmospheric MNPPs.^17,116,119^

## 6 Research Needs

Based on recent research advances, we outline the following research needs to understand atmospheric MNPPs:

- Identify sources and processes that lead to MNPPs emissions into the atmosphere: The increasing production of plastics suggests that new and emerging sources and processes will significantly contribute to the concentrations of atmospheric MNPPs. Many of these identified sources involve heat (incineration, pipe curing, and 3D printing). Human activities where plastic materials are exposed to heat should, therefore, be examined. Mechanical processes such as bubble bursting from turbulence in rivers (e.g., waterfalls), lakes, and wastewater treatment aeration tanks, as well as the resuspension of MNPPs from dust, plants, and other surfaces, and plastic surface abrasion, are additional MNPP sources that warrant further exploration.
- Develop consistent, reproducible, and accessible measurement methods: A continuing challenge is distinguishing MNPPs from naturally occurring polymers and identifying the often very low concentrations of MNPPs in atmospheric samples. Key challenges for MNPP microspectroscopy to overcome are complicated workflows, reproducibility issues, and chemical composition. For both microspectroscopy and mass spectrometry analysis of MNPPs, incorporating machine learning and other automated methods may provide streamlined and cost-effective analysis.
- Assess the relative importance of primary vs secondary sources: Secondary processes, such as the fragmentation of atmospheric MPPs into NPPs, are more difficult to characterize but can contribute to many more NPPs, especially in terms of number or surface area. Understanding the kinetics and mechanisms of fragmentation in the atmosphere will allow for better estimations.
- Reconcile differences between top-down and bottom-up estimates of ocean emission fluxes: Laboratory experiments should better represent sea-to-air transfer by capturing the complexities at the sea surface, such as bubble scavenging and transfer into the sea surface microlayer and sea spray aerosol, and the effects of winds. More measurements are needed to improve the global coverage of MNPP measurements in the marine atmosphere.
- Mechanistic studies of NP aging: Controlled studies are needed to understand how atmospheric processing changes the chemical and physical properties of NPPs. These studies should be conducted under atmospherically relevant conditions, considering factors such as pH, ionic strength, organic matrix, oxidants, particle size, and air–particle interface that are specific to atmospheric particles.
- Additives and organic matrices: Plastic materials often contain a multitude of chemical additives, and studies are needed on the rates and mechanisms of their leaching and aging. Studies on the sorptive partitioning of chemicals into and out of NPPs, therefore, need to consider conditions unique to atmospheric particles.
- Aerosol microphysics of MNPPs: More studies are needed on the hygroscopic properties of NPPs, especially aged NPPs, which may contain more hydrophilic functional groups. As more is understood about the CCN and ice nucleating activity of MNPPs, modeling studies are needed to estimate their global climate impacts.
- Toxicity of MNPPs: Although many plastic materials are considered sterile and safe, chemical additives have shown adverse impacts on human and ecosystem health. It is, therefore, imperative that future studies around health impacts distinguish between the effects of polymers and those from additives. Non-targeted analysis should also be performed to understand what additives are used in different plastic materials. A more nuanced understanding of toxicity will help policies around chemical use and guide future material design to minimize health impact.

These research needs still indicate large knowledge gaps concerning atmospheric MNPPs. Specifically, the concentration, source, and distribution of atmospheric MNPPs are largely unknown, which inhibits a comprehensive understanding of their transport, chemical processing, and physical loss. As plastic debris has increased substantially coincident with its production, the atmospheric portion of MNPPs is also expected to increase significantly in upcoming decades, further increasing potential effects on climate and health. Systematic studies are urgently needed to better constrain the atmospheric properties, transformation, and impacts of MNPPs.
